# Lumbar Epidural Hematoma as a Rare Complication From Minimally Invasive Lumbar Decompression

**DOI:** 10.7759/cureus.51083

**Published:** 2023-12-25

**Authors:** Samuel A Tenhoeve, Michael Karsy

**Affiliations:** 1 Neurosurgery, University of Utah, Salt Lake City, USA; 2 Neurosurgery, Global Neurosciences Institute, Philadelphia, USA; 3 Neurosurgery, Drexel University College of Medicine, Philadelphia, USA

**Keywords:** spinal decompression, acute spinal hematoma, chronic low back pain (clbp), lumbar spinal stenosis (lss), multi-modality pain management, inter-professional practice, minimally invasive surgical procedures, minimally-invasive spine, lumbar epidural hematoma, minimally invasive lumbar decompression

## Abstract

Lumbar spinal stenosis (LSS) is a common and debilitating pathology globally. Conservative and surgical treatment options exist for patients. Recently, minimally invasive lumbar decompression (MILD) has been described as a less invasive technique for the treatment of early spinal stenosis ≥2.5mm ligamentum thickening or in patients at high risk for general anesthesia. Often, MILD is performed by interventional pain providers and shows low complication rates. We describe a 76-year-old woman who presented to the emergency department immediately after undergoing a MILD procedure at an outside surgery center with lower back/sacral pain resulting from an acute epidural hematoma extending from T12-L3. Early recognition and surgical evacuation resulted in a good outcome with no complications. Our goal is to increase awareness of this rare complication and encourage multidisciplinary approaches to managing LSS between spine surgeons and pain providers.

## Introduction

*Chronic low back pain* is typically defined as pain concentrated in the lumbosacral region of the spine that lasts longer than three months [1]. There is a high likelihood that >60% of people will experience debilitating low back pain during their lifetime, with an increased prevalence in populations of advanced age, low socioeconomic status, and those with more comorbidities [2,3]. Among the spectrum of causes for lower back pain, lumbar spine stenosis (LSS) occurs with an incidence of up to 38% globally [4]. The North American Spine Society has formally defined LSS as "a condition in which there is diminished space available for the neural and vascular elements in the lumbar spine secondary to degenerative changes in the spinal canal" [5].

This spinal canal narrowing can result from osteoarthritis, degenerative disk disease, ligamentum hypertrophy, and generalized joint degeneration [6-9]. The evidence suggests that for many patients, a multimodal approach to treatment is the most suitable, including conservative and surgical options, ranging from analgesic pain medications to lumbar fusion surgeries [10,11].

First described in 2005, the Minimally Invasive Lumbar Decompression (MILD) procedure has become one of the mainstay treatments when ligamentum flavum hypertrophy is present (Vertos Medical, Aliso Viejo, CA, USA) [12]. The procedure is carried out under sedation and through percutaneous access for patients with symptomatic LSS and ligamentous hypertrophy ≥2.5mm. Contraindications include patients with grade II spondylolisthesis. Debulking of hypertrophic ligamentum flavum and the corresponding interlaminar bone is the primary goal. This localized removal of the canal-narrowing pathology allows spinal cord decompression at the intended target [13].

Prospective studies, case reports, and extensive review articles have assessed the safety and efficacy of the procedure [12-15]. In a recent review, Jain et al. detailed that two level-one randomized control trials, five prospective studies, two meta-analyses, four retrospective studies, and three case series had been published justifying the safety profile of the MILD procedure. Of these studies, only Staats et al. reported a 1.3% device- or procedure-related adverse event among its 143 patients treated with the MILD procedure, while none noted major complications such as dural tear, cerebrospinal fluid leak, or wound-healing complications. They also showed a significantly low likelihood of reoperation, 5.6% [5,16].

We report a case of a rare epidural hematoma forming after a MILD procedure and the multidisciplinary approach to diagnosis and treatment.

## Case presentation

A 76-year-old female patient with a past medical history of osteoarthritis and coronary artery disease presented to the emergency department after having undergone a left L2/L3 MILD procedure under monitored anesthesia care that same day for degenerative spondylosis and moderate-to-severe LSS. She denied antiplatelet use or a history of bleeding disorder. Upon discharge following the procedure, she had worsening lumbar and left buttock/hip pain worsened with movement, left leg radiculopathy, lower extremity weakness, and progressive bilateral numbness of her lower extremities. She denied bowel/urinary incontinence, saddle paraesthesia, and signs of post-operative infection. Her family and social histories were found to be unremarkable for the current presentation, and she denied any pertinent medication use, including anticoagulation and antiplatelet products.

Imaging findings

**Figure 1:**
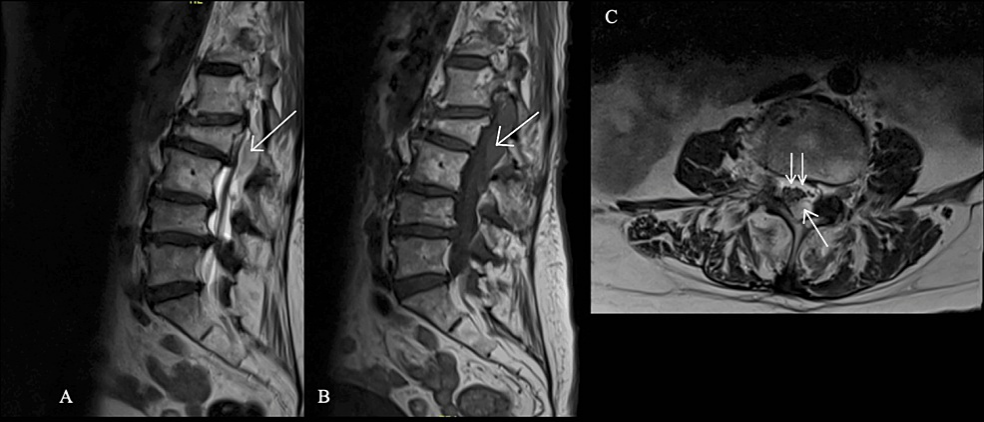
Imaging demonstrating a dorsal epidural hematoma after MILD procedure A) Sagittal T2 imaging demonstrating a large dorsal, mix-density fluid collection (arrow) with compression of the thecal sac. B) Sagittal T1 demonstrating some T1 shortening of a dorsal epidural hematoma (arrow). C) Axial T2 imaging demonstrates a left eccentric dorsal epidural hematoma (arrow) with compression and rightward displacement of the thecal sac (double arrow).

The presenting symptoms prompted further investigation using radiographic imaging consisting of computed tomography (CT) and magnetic resonance imaging (MRI). The observations from both techniques indicated a large dorsal epidural collection spanning from the T12 to L3 vertebrae with compression of the thecal sac. The imaging also noted persistent multilevel central canal and neuroforaminal stenosis (Figure 1).

 Surgical approach

The patient was treated with perioperative corticosteroids and analgesia for pain control. Due to the acuity of the hematoma and its significant size, she was recommended for surgical evacuation. After proper sedation and positioning, fluoroscopy was used to localize the L1 to the interspinous site. Baseline neuromonitoring sensory- and motor-evoked potentials were obtained. This planned incision corresponded well with the entry point for the MILD procedure in the midline. The prior left side paramedian incision was infiltrated with half percent lidocaine containing epinephrine and opened. A second fluoroscopy shot was used to localize the L1-2-disc space. A laminectomy at L1 was performed widely. Straight curettes were then used to dissect into the epidural space, and Kerrison rongeurs were used to remove thickened ligamentum flavum out to the medial facet. We identified a clot eccentric to the left side, which displaced the cord towards the right. We evacuated the clot and also used a Woodson dissector and flexible Angiocath to further irrigate superiorly and inferiorly. The thecal sac rebounded back to normal position well. Neuromonitoring improved on the patient's right side after the clot evacuation. A surgical drain was left for 24 hours. Postoperatively, the patient showed improved strength and sensation in the lower extremities and reduced radicular pain. At one and three-month follow-ups, she had returned to her neurological baseline.

## Discussion

Given the significant prevalence of LSS, additional modalities for treatment, such as the MILD procedure, can be effective approaches for patients [7]. In addition, surgical risks from anesthesia are reduced for patients undergoing mild procedures as they can be performed under monitored anesthesia care. While complication rates for the MILD procedure are rare, we describe a case of an epidural hematoma after such a procedure where early recognition and treatment resulted in a good patient outcome. We postulate that the hematoma was a result of unrecognized and uncontrolled bleeding due to the minimally invasive nature of the procedure. Both spine surgeons and pain physicians should be aware of the indications, complication profile, and management of patients who undergo the MILD procedure.

Multiple studies have supported the safety and efficacy of the MILD procedure [12-15]. In 2020, Jain et al. reviewed two randomized controlled trials and eleven other clinically controlled studies and determined that when proper conservative treatment methods precede the MILD procedure, it is a safe and effective treatment. Additionally, they described that patients should demonstrate symptomatic LSS, have imaging confirmation of spinal stenosis, and have a ligamentum flavum hypertrophied with a thickness of at least 2.5 mm for consideration. Finally, any patient with prior spine surgery or a current localized infection at the level of operation should be excluded, while those with higher than grade II spondylolisthesis, bleeding diatheses/coagulation disorder, and the presence of a systemic infection are all relative contraindications [5]. In 2016, Benyamin et al. reported on the MiDAS ENCORE randomized control trial that followed 149 patients who underwent the MILD procedure throughout twenty-six pain centers in the United States. This treatment group was compared to a control group that received ultrasound-guided lumbar interlaminar epidural steroid injections (ESI) using the Oswestry Disability Index (ODI), Numeric Pain Rating Scale (NPRS), and all three Zurich Claudication Questionnaire (ZCQ) domains. At the 6-month follow-up, 62.2%, 55.9%, and 52.8% of the MILD group experienced improvement in the ODI, NPRS, and ZCQ domains, respectively. These findings demonstrated a statistically significant difference between the ESI group that reported percentages of 35.7%, 33.3%, and 28.7% for those same measures. This trend held through the 1-year follow-up with comparisons of 58.0% vs. 27.1%, 57.3% vs. 27.1%, and 51.7% vs. 31.8%, ODI, NPRS, and ZCQ measures [17]. Later, in 2018, Staats et al. declared these findings held at the 2-year follow-up, even going further to show that only eight of the remaining 143 patients from the treatment group had undergone a further surgical procedure, 22 had received an ESI or nerve block, one underwent a rhizotomy, and one received an intrathecal pump [16]. These results were supported by a larger review by Kaye et al. in 2021, which covered the different minimally invasive procedures available for treating LSS and degenerative disk disease [15]. Their review shows that the MILD can be an effective treatment option for those patients who meet the indications for surgery.

Despite these findings, there have been reports of serious post-operative complications. Tumialán et al. described serious complications in response to a study by Mekhail et al., including eight patients with refractory neurogenic claudication and two patients with CSF leak. One patient with a CSF leak also experienced an incidental dural tear and transection of spinal nerve roots and never regained baseline function [18,19]. While this is a single-center response to the possible post-operative complications, this letter was written in 2012 and highlighted the need for more current and detailed post-operative outcome reporting. At the 2-year follow-up of the ENCORE trial in 2018, Staats et al. reported that one of the operations, "oozing," was present at the site of decompression. They subsequently injected Gelfoam through the surgical cannula into the interlaminar space, and the patient recovered uneventfully.

Additionally, one patient during the trial experienced post-operative pain correlated with the MILD procedure that was relieved within 3 days post-op [16]. Recently, Deer et al. established the guidelines for minimally invasive spine treatment. They agreed on eleven consensus points that guide the use of minimally invasive spine procedures using the 2011 Institute of Medicine (IOM) clinical practice guidelines. This framework required each point to be reconciled to established evidence, grade recommendations, and consensus levels among a group of nationally recognized spine experts [7].

As a consequence of reporting on this case, we hope that a critical part of that algorithmic approach will be consultation with an interdisciplinary team, including a spine surgeon, as this procedure is often carried out by individual pain management specialists in an outpatient setting. It is extremely well established that an interprofessional approach to treatment is beneficial for patient outcomes [20]. Additionally, all types of treatment modalities should be considered in preoperative planning, including but not limited to microscopic, exoscopic, and minimally invasive techniques. Further investigation is needed to identify the types of patients that could benefit the most from co-consultation with a spine surgeon during preoperative planning.

## Conclusions

There are many conservative and surgical approaches within the treatment possibilities of LSS. The MILD procedure does demonstrate benefit in selected patients with low complications. In the present report, we identify a serious complication from a MILD procedure in a patient who reported to the emergency department immediately postoperatively. We identify that interprofessional collaboration between pain physicians and spine surgeons will help optimize the selection of patients for MILD procedures and aid in the early detection and treatment of complications. Furthermore, we suggest conducting more comprehensive and updated reporting on minimally invasive procedures for LSS to help clinicians better guide patient treatment decisions.

## References

[1] Parthan A, Evans CJ, Le K (2006). Chronic low back pain: epidemiology, economic burden and patient-reported outcomes in the USA. Expert Rev Pharmacoecon Outcomes Res.

[2] Hartvigsen J, Hancock MJ, Kongsted A (2018). What low back pain is and why we need to pay attention. Lancet.

[3] Foster NE, Anema JR, Cherkin D (2018). Prevention and treatment of low back pain: evidence, challenges, and promising directions. Lancet.

[4] Jensen RK, Jensen TS, Koes B, Hartvigsen J (2020). Prevalence of lumbar spinal stenosis in general and clinical populations: a systematic review and meta-analysis. Eur Spine J.

[5] Jain S, Deer T, Sayed D (2020). Minimally invasive lumbar decompression: a review of indications, techniques, efficacy and safety. Pain Manag.

[6] Omidi-Kashani F, Hasankhani EG, Ashjazadeh A (2014). Lumbar spinal stenosis: who should be fused? an updated review. Asian Spine J.

[7] Deer TR, Grider JS, Pope JE (2019). The MIST guidelines: the lumbar spinal stenosis consensus group guidelines for minimally invasive spine treatment. Pain Pract.

[8] Abdul Rahman HAS, Iacob G (2015). General considerations in lumbar spinal stenosis. Rom Neurosurg.

[9] Deer T, Sayed D, Michels J, Josephson Y, Li S, Calodney AK (2019). A review of lumbar spinal stenosis with intermittent neurogenic claudication: disease and diagnosis. Pain Med.

[10] Stochkendahl MJ, Kjaer P, Hartvigsen J (2018). National clinical guidelines for non-surgical treatment of patients with recent onset low back pain or lumbar radiculopathy. Eur Spine J.

[11] Xu W, Ran B, Luo W, Li Z, Gu R (2021). Is lumbar fusion necessary for chronic low back pain associated with degenerative disk disease? a meta-analysis. World Neurosurg.

[12] Racz GB, Heavner JE, Bosscher H, Helm S 2nd (2013). The MILD procedure. Pain Pract.

[13] Chen H, Kelling J (2013). mild procedure for lumbar decompression: a review. Pain Pract.

[14] Schomer DF, Solsberg D, Wong W, Chopko BW (2011). mild(®) lumbar decompression for the treatment of lumbar spinal stenosis. Neuroradiol J.

[15] Kaye AD, Edinoff AN, Temple SN (2021). A comprehensive review of novel interventional techniques for chronic pain: spinal stenosis and degenerative disc disease-MILD percutaneous image-guided lumbar decompression, vertiflex interspinous spacer, minuteman G3 interspinous-interlaminar fusion. Adv Ther.

[16] Staats PS, Chafin TB, Golovac S (2018). Long-term safety and efficacy of minimally invasive lumbar decompression procedure for the treatment of lumbar spinal stenosis with neurogenic claudication: 2-year results of MiDAS ENCORE. Reg Anesth Pain Med.

[17] Benyamin RMS, P. S (2016). MILD® is an effective treatment for lumbar spinal stenosis with neurogenic claudication: MiDAS ENCORE randomized controlled trial. Pain Physician.

[18] Tumialán LM, Marciano FF, Theodore N (2012). Regarding: long-term results of percutaneous lumbar decompression mild for spinal stenosis. Pain Pract.

[19] Mekhail N, Vallejo R, Coleman MH, Benyamin RM (2012). Long-term results of percutaneous lumbar decompression mild(®) for spinal stenosis. Pain Pract.

[20] Heip T, Van Hecke A, Malfait S, Van Biesen W, Eeckloo K (2022). The effects of interdisciplinary bedside rounds on patient-centeredness, quality of care, and team collaboration: a systematic review. J Patient Saf.

